# Precipitation Changes in the Three Gorges Reservoir Area and the Relationship with Water Level Change

**DOI:** 10.3390/s21186110

**Published:** 2021-09-12

**Authors:** Qin Li, Xiuguo Liu, Yulong Zhong, Mengmeng Wang, Manxing Shi

**Affiliations:** 1 School of Geography and Information Engineering, China University of Geosciences, Wuhan 430078, China; liqin@cug.edu.cn (Q.L.); liuxiuguo@cug.edu.cn (X.L.); wangmm@cug.edu.cn (M.W.); shimanxing@cug.edu.cn (M.S.); 2 Artificial Intelligence School, Wuchang University of Technology, Wuhan 430223, China

**Keywords:** precipitation, impoundment, water level, Three Gorges Project (TGP), Precipitation Concentration Degree (PCD), Precipitation Concentration Period (PCP)

## Abstract

As the largest hydroelectric project worldwide, previous studies indicate that the Three Gorges Dam (TGD) affects the local climate because of the changes of hydrological cycle caused by the impounding and draining of the TGD. However, previous studies do not analyze the long-term precipitation changes before and after the impoundment, and the variation characteristics of local precipitation remain elusive. In this study, we use precipitation anomaly data derived from the CN05.1 precipitation dataset between 1988 and 2017 to trace the changes of precipitation before and after the construction of the TGD (i.e., 1988–2002 and 2003–2017), in the Three Gorges Reservoir Area (TGRA). Results showed that the annual and dry season precipitation anomaly in the TGRA presented an increasing trend, and the precipitation anomaly showed a slight decrease during the flood season. After the impoundment of TGD, the precipitation concentration degree in the TGRA decreased, indicating that the precipitation became increasingly uniform, and the precipitation concentration period insignificantly increased. A resonance phenomenon between the monthly average water level and precipitation anomaly occurred in the TGRA after 2011 and showed a positive correlation. Our findings revealed the change of local precipitation characteristics before and after the impoundment of TGD and showed strong evidence that this change had a close relationship with the water level.

## 1. Introduction

As an essential infrastructure, dams provide numerous conveniences for people’s lives and production and contribute considerably to economic development [1]. In addition to their enormous societal benefits, more land is converted to surface water with the construction of a dam. This change can lead to the increased availability of local moisture and significantly affect mesoscale circulation [2,3]. Generally speaking, the mesoscale circulation is basically a “local” ranging from 10 km to 100 km, and one of the local effects on this change can be the modification of precipitation [4].

The Three Gorges Project (TGP) is one of the world’s largest and most functional hydroelectric hub projects. The Three Gorges Dam (TGD) brings great social and economic benefits, such as electricity generation, flood control, and shipping access [5]. Moreover, the increase of the underlying surface area of the water body and the climate change of the large-scale background field leads to changes in the frequency and characteristics of local meteorological disasters [1,6,7]. The influence of TGD on local climate and the probable effect on precipitation patterns has attracted wide attention.

Some studies have suggested that the impoundment of TGD can slightly affect local meteorological conditions, such as temperature and precipitation in the Three Gorges Reservoir Area (TGRA) [8,9,10,11,12,13]. However, other studies have proposed different conclusions. Wu et al. [14] showed that the impoundment of TGD led to a decrease in precipitation near the dam and an increase in precipitation to the north and west of the dam, which may affect the climate on a regional scale. Fang et al. [15] discovered significant decreases in spring, fall, winter, and annual number of rainy days and significant increases in precipitation intensity in the TGRA.

There may be several reasons for the different findings in previous studies. First, most of the precipitation data used in previous studies have been achieved by satellite. Satellite observation has some advantages, such as wide spatial coverage, uninhibited by terrain, and continuous observation [16,17]. However, satellite-based precipitation data are measured indirectly, which is easily limited by cloud characteristics and retrieval algorithms. The accuracy of precipitation monitoring still needs improvement [18,19,20]. Second, most of the previous studies only analyzed a relatively short time period, which did not cover the entire impounding period of TGP from 2003 to 2010 and the stable operation period after 2011, and the long time-series precipitation changes before 2003 were also not considered [11,12,14,15]. Therefore, the short period limits the reliability and accuracy of the results and conclusion. Third, previous scholars have focused on annual and seasonal changes in precipitation, without considering the variation of the time and degree of concentration of the yearly total precipitation within a year. However, since the 1990s, there has been a high frequency of flood occurrence in the Yangtze River due to the increase of intense precipitation events and the consequent increase in their degree of precipitation concentration. The changes in spatial and temporal concentration degree and variation of precipitation in the Yangtze River were the major possible reasons for the frequent flood disasters [21]. Higher precipitation concentration, represented not only by higher percentages of the annual total precipitation in a few extremely rainy days, but also by the time and degree of concentration of the yearly total precipitation within a year, may lead to floods and droughts, which are expected to put considerable pressure on water resources [22]. Zhang et al. (2003) [22] defined precipitation concentration period (PCP) and precipitation concentration degree (PCD). The PCP represents the period in which the total precipitation within a year concentrates and the PCD represents the degree to which annual total precipitation is distributed in a year. The PCP and PCD can quantitatively analyze the basic characteristics and formation mechanism of drought and flood disasters and are widely used to analyze of temporal changes in precipitation characteristics [23,24,25]. Therefore, understanding the changes in PCD and PCP is important to analyze the effect of the TGP on regional climate and the environment. Finally, since the TGD was put into use, the water level in the reservoir area has constantly been changing, but few studies have focused on the relationship between water level and precipitation.

In the late 1980s, data assimilation techniques were proposed to reconstruct historical forcing data at high resolution [26]. The resulting forcing datasets are widely used because they have high precision, wide coverage, long time span, and easy access and processing. [27,28,29,30]. Among these forcing datasets, the CN05.1 forcing dataset has been developed and maintained by Chinese scientists and is supposed to be accurate and reliable because surface observations more strictly constrain it [31]. Although CN05.1 has high accuracy in the whole Chinese region, it needs further confirmation when used in local areas [32].

In this study, we use precipitation anomaly data derived from the CN05.1 precipitation dataset between 1988 and 2017 to study the changes of precipitation before and after the construction of the TGD in the TGRA.

Before we studied the changes of precipitation, the PCD, and the PCP before and after the impoundment in the TGRA. We confirmed the accuracy of the CN05.1 forcing dataset for the TGRA based on the precipitation station data provided by the Hubei Meteorological Bureau and other precipitation datasets. The remainder of this study is organized as follows. The study area and data are described in Section 2. The methods are presented in Section 3. The results are described in Section 4. The variation characteristics of precipitation in the TGRA and comparison with existing studies are discussed in Section 5. Finally, we provide a summary and give our conclusions in Section 6.

## 2. Materials

### 2.1. Study Area

**Figure 1:**
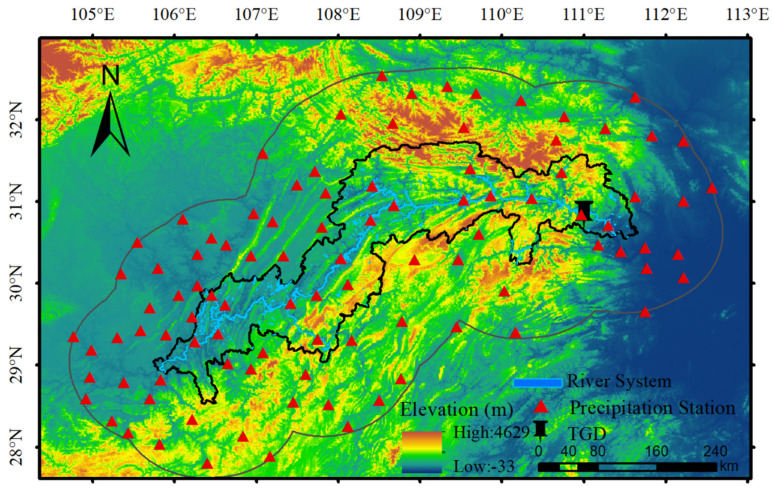
Study area with terrain. Black pin marks the Three Gorges Dam (TGD); the thick black line delineates the boundary of the Three Gorges Reservoir Area (TGRA); the thin black line represents the 1° buffer of the TGRA. Meteorological stations used in this study located within 1° buffer of the TGRA are marked as red triangles.

The TGRA refers to the area that flooded after the completion of the TGD (Figure 1). The TGRA is located between 28°31′ N–31°44′ N and 105°50′ E–111°40′ E. The range of elevation is −22 m to 2991 m. The length of the reservoir is about 660 km, and the average width is ~1.1 km. The reservoir can store 39.3 billion m^3^ water, which covers about 1062 km^2^. Most of the Yangtze River area from Chongqing to Yichang can be controlled by the TGP.

Since the TGD was put into operation in 2003, the water level has risen from 69 m to 175 m. In 2006, the designated level of 156 m was reached. In October 2010, the designed maximum impoundment level of 175 m was reached for the first time. Then, the water level remained at 175 m in the conventional stage. In November 2020, the TGP completed the overall completion and acceptance of all procedures. With the water level change, the water area changes accordingly, increasing from 408 km^2^ at the original level of 69 m to 453 km^2^, 718 km^2^, and 1062 km^2^ when the water level is at 135 m, 156 m, and 175 m, respectively [33].

### 2.2. Data

#### 2.2.1. CN05.1

In this study, we used the monthly precipitation data from CN05.1 for precipitation analysis. CN05.1 is a grid dataset with a resolution of 0.25° × 0.25° from 1961 to 2019 produced by the National Climate Center in China [31]. CN05.1 was produced by more than 2400 meteorological stations over China. The components of meteorological station equipment include meteorological sensors, meteorological collectors and transmission modules, power supply systems, protective boxes, brackets, etc. The function of a meteorological sensor is to monitor various meteorological elements, and a meteorological sensor corresponds to a meteorological monitoring element. The meteorological station mainly includes the following sensors: (I) Tipping bucket rain sensor; (II) Digital temperature and relative humidity sensor; (III) Analogue temperature sensor with negative temperature coefficient thermistor; (IV) Wind speed and direction sensor; (V) Two independent solar radiation intensity sensors [34,35].

The meteorological variables in the CN05.1 dataset include average temperature, precipitation, maximum temperature, minimum temperature, average wind speed, relative humidity and evapotranspiration. An “anomaly approach” was applied in the interpolation step. The spline surfaces were fitted as functions of latitude, longitude, and elevation. The elevation data used in the CN05.1 dataset was GTOPO30 data. GTOPO30 is based on data derived from eight sources of elevation information. Digital terrain elevation data (DTED) were used as the major source in China. DTED data was created from a variety of “best-available” sources, including measured data and remote sensor observation data. With the inclusion of more meteorological stations, the CN05.1 data are more reliable than the previous versions, which were based on about 700 meteorological stations [36].

#### 2.2.2. Station Precipitation Data

The ground meteorological station precipitation data used in our experiment are provided by the Hubei Meteorological Bureau. The dataset includes monthly precipitation data of 97 stations in a 1° buffer zone of the TGRA from 1988 to 2017. The seasonal and annual data are achieved by accumulating the monthly precipitation. Figure 1 shows the spatial distribution of these stations.

#### 2.2.3. Water Level Data

The daily water level data of the TGRA from 2003.5.1 to 2017.12.31 are provided by the Hubei Hydrology and Water Resources Center (http://slt.hubei.gov.cn/sw/ accessed on 7 July 2021). Monthly average water level data are calculated from daily water level data.

## 3. Methods

### 3.1. Accuracy Evaluation of Precipitation Datasets

In this study, validation against station-based observations is conducted by using a pixel-point method to pair station data with the gridded forcing data [32,37]. Two metrics are used to evaluate the accuracy of precipitation dataset: (1) The correlation coefficient (CC) can quantify the linear correlation between different grid datasets and station data, and (2) the root mean square error (RMSE) helps quantify the dispersion between different grid datasets and station precipitation data.

### 3.2. Precipitation Anomaly and Spatial Trend before and after the Impoundment

The precipitation changes in the TGRA include two parts. The first part is caused by local land surface and atmosphere changes. And the second part is due to large-scale climate impacts, such as El Niño or interdecadal oscillations [38,39,40]. To analyze the precipitation variations associated with the TGP, the effect of the TGP from large-scale climate impacts should be excluded. We used methods described in previous studies [14,33], which selected a regional background to represent the large-scale climate. In our study, the regional background is defined as the 1° spatial buffer from the TGRA (27.5° N–32.8° N, 104.8° E–112.7° E, as shown in Figure 1), which is consistent with the background region used by Wu et al. [14]. The effects of large-scale climate impacts are considered to be similar in the TGRA and its surrounding region. Therefore, we removed the large-scale climate impacts by subtracting the mean precipitation for the regional background of each month. After the above subtraction, a new time-series of precipitation anomaly was obtained to investigate the variations caused by local changes.

Precipitation characteristics were extracted based on the new time-series of precipitation anomaly. To highlight the influence on reservoir impoundment, the study was divided into two periods: the pre-impoundment period (1988–2002) and the post-impoundment period (2003–2017).

The Theil-Sen trend estimation was used in this study; Sen’s slope quantitatively assesses linear trends, as follows:

(1) $$\beta =Median\left(\frac{x_{j}-x_{i}}{j-i}\right)$$

where 1 < *i* < *j* < *n*, *n* is the size of the time series, and $x_{j}$ and $x_{i}$ are the time-series data of the trend to be analyzed. The positive slope represents an increasing trend, whereas the negative slope indicates a decreasing trend.

As the precipitation in the TGRA is usually concentrated in May to October, the spatial pattern of precipitation in different seasons is distinct. We divided a year into two periods. May–October belongs to the flood season, November–April (next year) compose the dry season [41].

### 3.3. Concentrated Characteristics of Precipitation

To analyze the concentrated characteristics of precipitation distribution in one year, the PCD and PCP defined by Zhang et al. [22] were used to represent the distribution characteristics of precipitation over time. Previous studies have shown that PCD and PCP methods can quantitatively reveal the non-uniformity of precipitation in the time field [42,43,44].

The fundamentals for calculating the PCD and the PCP are based on the vector of precipitation. The assumption is that precipitation is a vector quantity with both magnitudes and that the direction for a year can be seen as a circle (360°) (more details about this method can be found in Zhang et al. [22]). Then, the yearly PCP and PCD for a location can be defined as follows:

(2) $$PCD_{i}=\frac{\sqrt{R_{xi}^{2}+R_{yi}^{2}}}{R_{i}}$$

(3) $$PCP_{i}=\arctan(\frac{R_{xi}}{R_{yi}})$$

(4) $$R_{xi}=\sum_{j=1}^{N}r_{ij}*\sin\theta_{j}\;\cdot \;R_{yi}=\sum_{j=1}^{N}r_{ij}*\cos\theta_{j}$$

where *i* is the year (*i* = 1988, 1989, …, 2017), *j* represents the hou (a year is divided into 72 hou and a month into 6 hou, ~5 days is defined as 1 hou) in a year (*j* = 1, 2, …, 72), $r_{ij}$ denotes total precipitation in the *j*th hou in the *i*th year, $\theta_{j}$ is the azimuth of the *j*th hou, and $R_{i}$ is the total precipitation of the station/grid in year *i*.

### 3.4. Cross-Wavelet Transform (CWT) Analysis

Hydrometeorological time series have characteristics of randomness, ambiguity, non-linearity, non-stationarity, and multiple time scales. CWT can analyze the time-frequency domain fluctuations of two mutually coupled time series based on wavelet transform. CWT decomposition of hydrometeorological data for multiscale analysis has been widely used in recent years [45,46]. Sun et al. [47] used the CWT to analyze the relationship between precipitation and water storage in southwestern China.

This study explores the possible relationship between the precipitation anomaly and monthly average water level by CWT analysis. Grinsted et al. [48] described the specific algorithm in detail. The MATLAB CWT toolbox can be download from GitHub (available at https://github.com/grinsted/wavelet-coherence/ accessed on 7 July 2021).

## 4. Results

### 4.1. Accuracy of CN05.1 Dataset in the Three Gorges Reservoir Area (TGRA)

**Figure 2:**
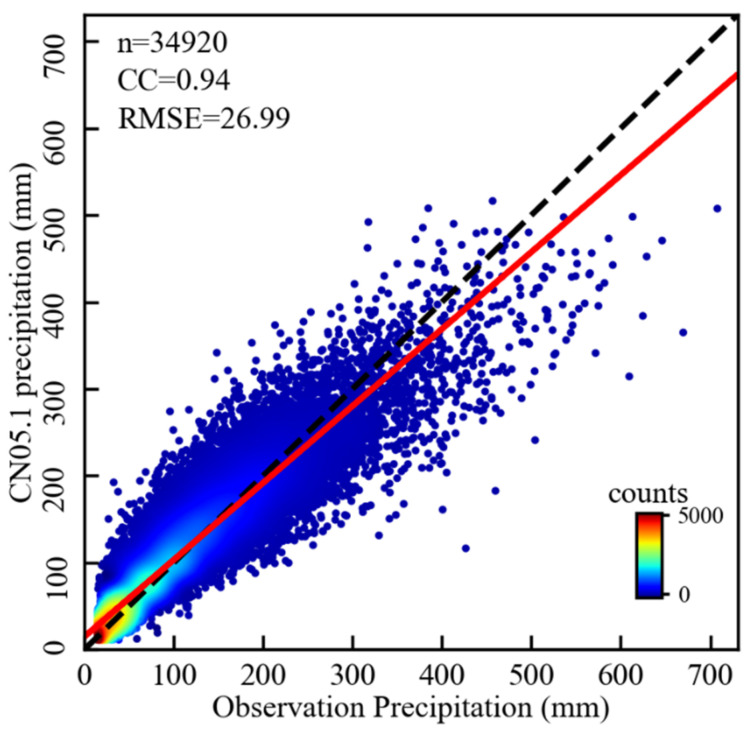
Density scatterplots of the monthly precipitation from CN05.1 versus ground-based observation. The black dashed line is the 1:1 line. n: number of valid samples; CC: Pearson correlation coefficient; RMSE: root-mean-square error of the monthly precipitation (mm); The color-coded dots represent the number of samples.

As shown in Figure 2, monthly estimates of precipitation from January 1988 to December 2017 are consistent with the Hubei Meteorological Bureau observations according to the CC and RMSE metrics. To further analyze the accuracy of the CN05.1 in the 1° buffer zone of the TGRA, we calculated the CC and RMSE among the CN05.1 and the observation data in different months during the study period. Table 1 shows the results. CN05.1 provides reliable precipitation data in most months of the year during the study period. In terms of average monthly precipitation for different months, the difference between CN05.1 and station observations is small. And we also evaluated the accuracy of two other precipitation forcing data of China in the TGRA, refer to Appendix A. Thus, it is reliable to select CN05.1 data to study the precipitation changes before and after the impoundment of the TGP.

### 4.2. Analysis on the Variation of Precipitation Anomaly and Its Spatial Trend

**Figure 3:**
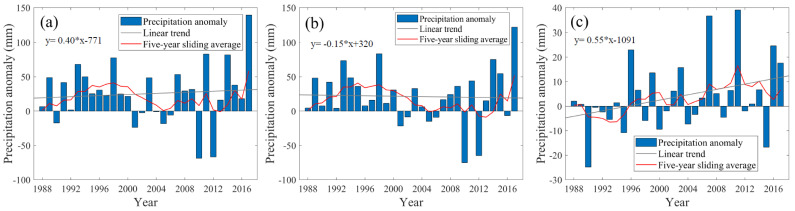
Change of precipitation for the (**a**) whole year, (**b**) flood season, and (**c**) dry season in the TGRA from 1988 to 2017.

Figure 3 displays the annual and seasonal precipitation anomaly in the TGRA from 1988 to 2017. The annual precipitation anomaly shows an increasing trend during the whole study period, with a linear trend of 0.40 mm/yr, but it does not pass the significance test (*p* value < 0.05). The maximum precipitation anomaly is 139.24 mm, which is in 2017. The minimum precipitation anomaly is −68.60 mm and occurs in 2010. The precipitation anomaly in the flood season during the study period shows a decreasing trend (Figure 3b), with a linear trend of −0.15 mm/yr, the linear trend does not pass the significance test (*p* value < 0.05). The maximum value of precipitation anomaly occurs in 2017, which is 121.65 mm. The minimum value is −75.02 mm in 2010. The maximum and minimum values of precipitation anomaly in the flood season occur in the same year as the annual precipitation anomaly. The precipitation anomaly in the flood season could explain most of the annual precipitation anomaly in the two years. The decrease of precipitation in the flood season may be related to the increase of water surface temperature in summer. It leads to weaker updrafts and more underdrafts, which dissipate water vapour in the planetary boundary layer [12].

**Figure 4:**
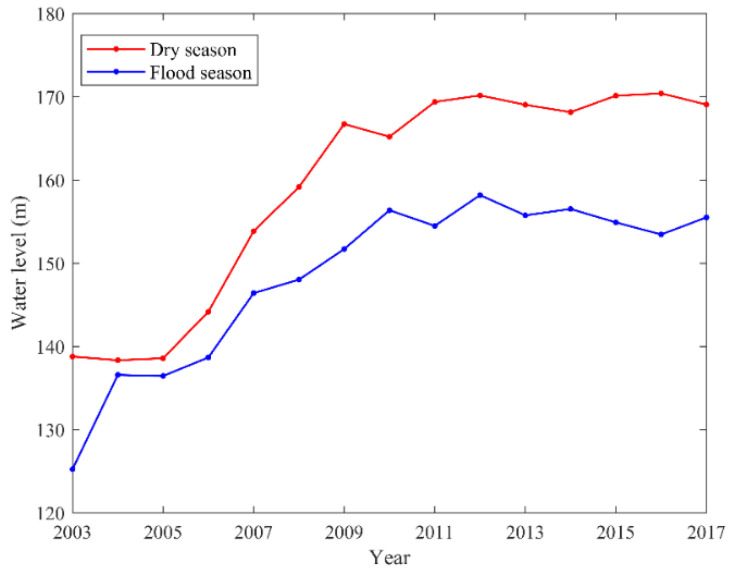
Variation of monthly average water level in dry and flood seasons from 2003 to 2017 in the TGRA.

Figure 3c shows the change in precipitation anomaly in the dry season. The precipitation anomaly has an increasing trend during the whole study period (*p* value > 0.05). The maximum value of precipitation anomaly is in 2011 (39.03 mm), and the minimum value is in 1990 (−24.82 mm). The impoundment of the TGRA generally began in November, corresponding to the dry season in our study. The measured water level data of the Three Gorges Reservoir station from 2003 to 2017 (Figure 4) also shows that the water level in the dry season of the TGRA is greater than that in the flood season. After the impoundment of the TGRA, the water area increases. This change affects the local water vapor cycle from small scale [49], which may be the reason for the increase of precipitation anomaly in the dry season.

**Figure 5:**
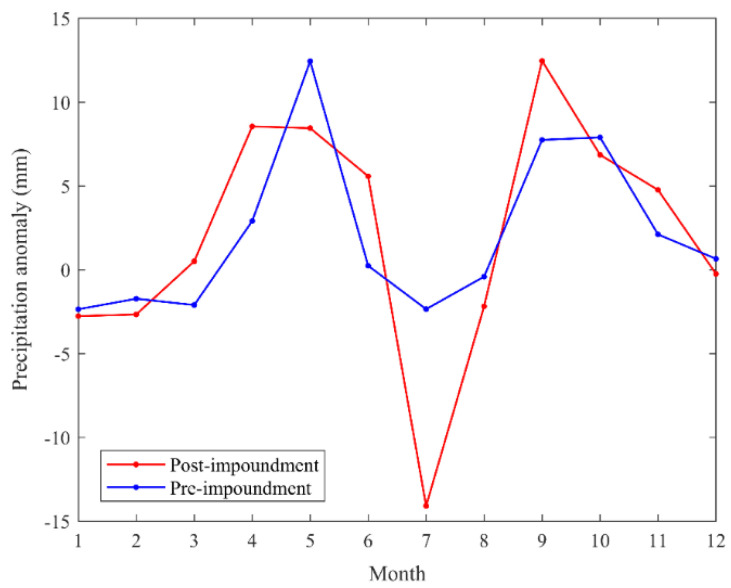
Monthly precipitation anomaly before and after the impoundment in the TGRA.

Figure 5 shows the time series of monthly precipitation anomaly before and after the impoundment (refer to the monthly average precipitation anomaly in the two periods from 1988 to 2002 and 2003 to 2017). The result shows that July has the largest difference in precipitation anomaly before and after the impoundment in the year. The precipitation is 11.74 mm less than that before the impoundment. This result is consistent with the decreasing trend of the precipitation anomaly in the flood season analyzed in the previous section. July is the month with the most precipitation in the Yangtze River Basin, but the average precipitation anomaly in July is the smallest in all months in the year after the impoundment. Impoundment and drainage affect the precipitation anomaly in the TGRA.

**Figure 6:**
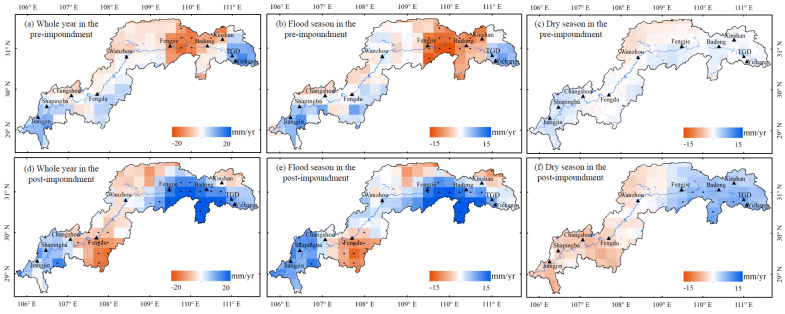
Distribution of precipitation anomaly trends before the impoundment for the (**a**) whole year, (**b**) flood season, (**c**) dry season, and after the impoundment for the (**d**) whole year, (**e**) flood season, and (**f**) dry season; the black point stands for that the pixel has passed the significance test (*p* value < 0.05).

Figure 6 displays the spatial distribution of annual and seasonal precipitation anomaly trends before and after the impoundment in the TGRA. As shown in Figure 6a–c, the annual and flood season precipitation anomaly decreased in the area between Badong and Fengjie Stations before the impoundment. The region near Yichang Station shows a trend of becoming wet. The precipitation anomaly in the dry season before the impoundment does not show a clear trend.

As shown in Figure 3b, in the flood season, although the precipitation anomaly shows a decreasing trend from 1988 to 2017, the precipitation anomaly shows an increasing trend from 2003 to 2017. Therefore, after impoundment, the spatial distribution of annual, flood season, and dry season precipitation anomaly is dominated by an increasing trend. Figure 6d–f demonstrate that in the annual, flood, and dry season, precipitation anomaly around the TGD shows a different increasing trend, especially in the area between Badong and Fengjie Stations. The precipitation anomaly also greatly changed in the southern part of Fengdu Station in the upper reaches of the TGRA, which decreased after the impoundment in the annual, flood, and dry season. No obvious change is observed in other regions before and after the impoundment.

Overall, the region near the dam shows the largest variation in precipitation anomaly, which may be related to the largest variation of water area around the dam [50]. The precipitation in the flood season is higher than that in the dry season. Therefore, the change pattern of annual precipitation anomaly is mainly affected by the change in precipitation in the flood season. In the dry season, the main change is that precipitation anomaly decreased in the west and increased in the east after the impoundment. The changes are smaller than that in a whole year and the flood season.

### 4.3. Variation of Precipitation Concentration Degree (PCD) and Precipitation Concentration Period (PCP) in the TGRA

#### 4.3.1. Time-Series Variation of PCD and PCP

**Figure 7:**
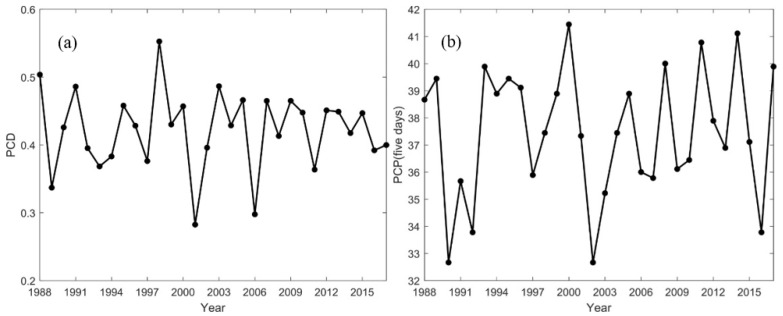
Interannual variation of (**a**) precipitation concentration degree (PCD) and (**b**) precipitation concentration period (PCP) in the TGRA from 1988 to 2017.

Figure 7 shows the interannual variations of PCD and PCP in the TGRA from 1988 to 2017. In the TGRA, the average PCD value over 30 years is 0.45 (Figure 7a). The interannual changes are drastic. The minimal value for the whole study period is 0.28 (in 2001). The maximal value of PCD is 0.54 (in 1998). The PCD values change obviously in different stages (the pre-impoundment and the post-impoundment). From 1988 to 2002, the average value of PCD is 0.48. PCD fluctuates greatly, and the standard deviation is 0.07. The change is more stable from 2003 to 2017, the average value of PCD is 0.42, and the standard deviation is 0.05. The precipitation becomes more uniform throughout the year than that before the impoundment. For the whole study period, the years with high PCD values are 1988, 1991, 1998, and 2003; and the years with low PCD values are 1989, 2001, and 2016. Generally, if the years have a high precipitation anomaly and a high PCD value, serious flood disasters would occur, such as the catastrophic flood disaster in the Yangtze River Basin in 1998. If the years have a high precipitation anomaly but with a low PCD value, then flood disasters are less likely to happen.

The mean of PCP time series is 37.49 hou in the TGRA (Figure 7b), mainly concentrated in mid-July. The minimal value is 32.61 hou (in 1990), and the maximal value is 41.82 hou (in 2000). PCP shows similar changes with PCD. The standard deviation of PCD is 2.72 in the pre-impoundment and 2.24 in the post-impoundment. Thus, the value changes sharply before the impoundment. After the impoundment, the value of PCP fluctuates slightly. The standard deviation of PCP is 1.23 in the pre-impoundment and 1.01 in the post-impoundment. The average value of PCP in the pre-impoundment is 37.38 hou. The average value in the post-impoundment is 37.59 hou. Thus, PCP is delayed relative to that before the impoundment.

#### 4.3.2. Spatial Pattern of PCD and PCP in the TGRA

**Figure 8:**
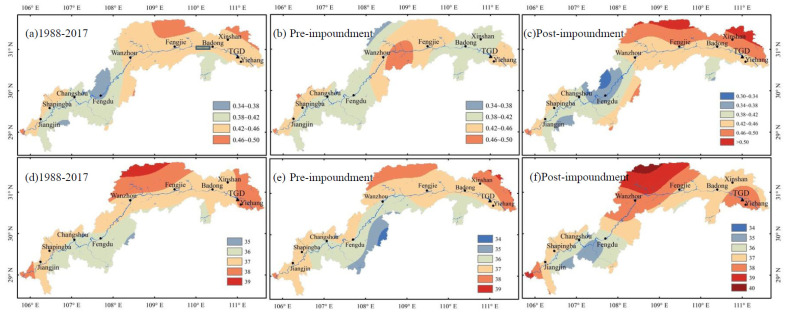
Spatial distribution of (**a**) PCD from 1988 to 2017, (**b**) PCD before the impoundment, (**c**) PCD after the impoundment, (**d**) PCP from 1988 to 2017, (**e**) PCP before the impoundment, and (**f**) PCP after the impoundment in the TGRA.

Figure 8 reveals the spatial distribution of PCD and PCP in the TGRA before and after the impoundment from 1988 to 2017. Figure 8a–c show that the PCD of the TGRA varied between 0.34 and 0.50 during the whole study period. The areas with larger PCD are mainly concentrated in the downstream of the TGRA. The comparison between Figure 8b,c shows that the PCD varies greatly along the downstream basin below Wanzhou Station before the impoundment. The PCD ranges from 0.42 to 0.46 after the impoundment. The area of PCD increase is mainly in the north of the downstream of the TGRA. The area where PCD shows a decreasing trend is mainly around Fengdu Station.

In Figure 8d–f, the PCP shows a decreasing trend from north to south during the whole study period. During the entire study period, the PCP of the TGRA is mainly between 35 hou and 39 hou. The PCP before the impoundment is between 34 hou and 39 hou. After the impoundment, the PCP in most areas is delayed by 1 hou to 2 hou compared with that before the impoundment. The obvious change is that the area in the TGD and around Yichang Station is delayed from 37 hou to 38 hou after the impoundment. The middle and northern of the TGRA is postponed from 37 hou to 39–40 hou after the impoundment.

On the basis of the above analysis, the PCD and PCP show two important characteristics. (1) After the impoundment, the spatial distribution of PCD has a large variation, and the range of PCD value is also larger than before the impoundment. (2) The contour line is basically in the direction of north-south. The PCD in the northeast is relatively large, and the PCD and PCP change greatly in the region near the TGD. The PCD and PCP also change greatly before and after the impoundment.

### 4.4. Relationship between Precipitation Anomaly and Water Level

**Figure 9:**
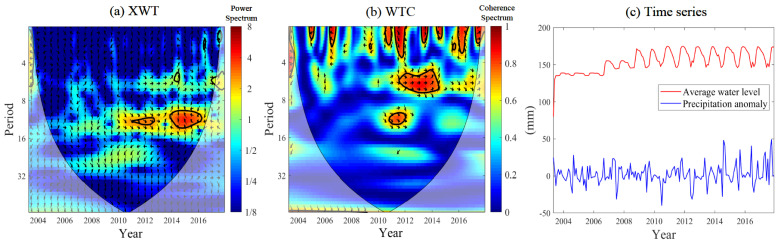
Wavelet coherence between precipitation anomaly and monthly average water level ((**a**) cross-wavelet power between precipitation anomaly and monthly average water level; (**b**) corresponding wavelet coherence between precipitation anomaly and monthly average water level; (**c**) time series of precipitation anomaly and monthly average water level).

To further explore the influence of the TGP on the variation of regional precipitation anomaly, CWT analysis is performed on the monthly precipitation anomaly and the monthly average water level. Figure 9a shows the cross-wavelet power between precipitation anomaly and monthly average water level in the TGRA. The monthly average water level and the monthly precipitation anomaly in the TGRA mainly exhibit significant resonance phenomenon after 2011. Moreover, there are 11–12 months and 10–14 months of resonance period, and the 95% red noise test is passed. The resonance period of 11–12 months is significant from 2011 to 2013, and 2014–2016 at 10–14 months based on the cross-wavelet power. The phase difference indicates that the water level change is ahead of the precipitation anomaly change.

The synchronization relationships between precipitation anomaly and the monthly average water level are further examined. Figure 9b shows the corresponding wavelet coherence between precipitation anomaly and monthly average water level in the TGRA. Based on the statistically significant regions identified in the WTC results, the water level changes have significant influences on precipitation anomaly in different periods in 2005–2017. The water level has the longest period influence on precipitation in the 5–7 months band. It also demonstrates that the monthly average water level and monthly precipitation anomaly exhibit obvious resonance phenomenon after 2010, and the resonance periods are mainly 12 months (2011–2012) and 5–7 months (2011–2014).

The monthly average water level and precipitation anomaly in the TGRA exhibit an obvious resonance phenomenon after 2011 (Figure 9). The relationship between the average water level and precipitation anomaly mainly exhibits a positive correlation. The influence of water level on precipitation anomaly in the TGRA mainly concentrated in the higher frequency, which is more obvious after 2011. The 175 m of experimental water storage in the TGRA was started in 2010, which reflects the natural regulation of precipitation in the TGRA by high water level operation. Overall, the improved understanding of the relationship between precipitation anomaly and the water level will also contribute to effectively investigate the future changes in precipitation in the TGRA.

## 5. Discussion

### 5.1. Variation Characteristics of Precipitation in the TGRA

After the impoundment of the TGD, the spatial precipitation pattern changed obviously, and the precipitation in the west of the TGD increased significantly. However, with the decrease of precipitation in the flood season and the increase of precipitation in dry season, the proportion of precipitation in dry season in the TGRA increased, which means that the seasonal distribution of precipitation was more uniform. With the completion of the impoundment of the TGR, the water area increased significantly, which caused a significant change of the relative humidity in the reservoir area [50], and this may be the main reason for the variation of the precipitation trends during the study period. In addition, after the impoundment, significant changes in land cover occurred in the TGRA [51]. The increase of spatial heterogeneity of precipitation in the TGRA may be associated with the increase of surface fragmentation, or the inconsistent response characteristics of different land cover to climate change [52].

More in-depth studies are needed to analyze the impact mechanism further. In the future, with the longer period of data accumulation, incorporating multi-source data into climate models would produce more evidence.

### 5.2. Comparison with Existing Studies

The climate effect of the TGP has been a continuous concern of the scientific community and the public. In 2006, Wu et al. [14] proposed that the climate effect of the TGP can occur on the scale of 100 km, the annual precipitation in the northwest of the TGRA increased significantly after 2003. However, Wu et al. [14] also pointed out that when using TRMM products for analysis, one of its major problems was uncertainty in data accuracy. Further studies over a longer period of time are needed to fully understand the effects of TGD on regional climate since Wu et al. [14] only covers 2006. In this study, CN05.1 with high accuracy in TGRA was used to analyze the change of precipitation anomaly from 1988 to 2017. Note that the annual precipitation anomaly shows an increasing trend, but the trend is not significant (*p* value > 0.05). The different results between this study and Wu et al. [14] may be resulted from the uncertainty of Wu et al. [14] using TRMM data and the short length of the analysis period.

In contrast, Xiao et al. [11] analyzed the variation of precipitation anomaly in TGRA from 1960 to 2005 by using the daily precipitation data of 27 stations in the TGRA. It was found that the monthly precipitation anomaly varied between −1 mm/day and 1 mm/day before and after the impoundment. Xiao et al. [11] concluded that the precipitation increase did not exceed the natural variability and the TGP had almost no effect on local precipitation. The data used by Xiao et al. [11] were only from 27 stations, and there was some limitation in using precipitation data from 27 stations to analyze the precipitation variation characteristics of the whole reservoir area. In addition, their research period only lasted until 2005.

**Figure 10:**
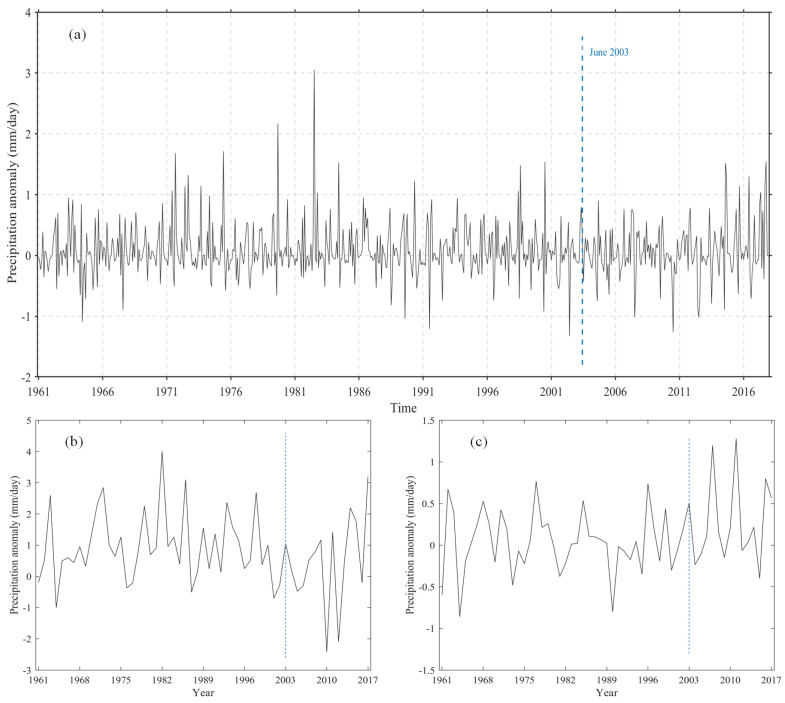
The change of (**a**) mean monthly precipitation anomaly, (**b**) flood season precipitation anomaly, and (**c**) dry season precipitation anomaly in the TGRA from 1961 to 2017.

To compare with the results of Xiao et al. [11], we analyzed the change of monthly precipitation anomaly in the TGRA from 1961 to 2017 according to their method, as shown in Figure 10a. It can be seen that the monthly precipitation anomaly in the TGRA does not increase or decrease significantly after 2003 (with a linear trend of 0.0012 mm/day), but we can find that the monthly precipitation fluctuation becomes smaller after 2003 (the standard deviation of precipitation anomaly before and after the impoundment are 0.44 and 0.42, respectively). During 1961–2017, the maximum and minimum monthly precipitation anomaly occurs before 2003. The difference between the maximum and minimum value of the monthly precipitation anomaly before 2003 is 4.34 mm/day, and the difference between the maximum and minimum value of the monthly precipitation anomaly after 2003 is 2.80 mm/day. We also calculate the precipitation anomaly of dry season and flood season in the TGRA from 1961 to 2017 (Figure 10b,c). As shown in this study, we suggest that the impact of the TGP on precipitation in the TGRA, i.e., the decrease of precipitation in the flood season, the increase of precipitation in the dry season, and precipitation within a year become uniform. This result is also confirmed by the PCD and PCP analyses. Xiao et al. [11] only analyzed the changes of monthly precipitation anomaly but did not analyze the changes of precipitation anomaly in the dry season and flood season. The flood season and dry season are distinct in the TGRA, so a comprehensive conclusion should be drawn from the analysis of multiple time scales.

## 6. Conclusions

In recent years, the impact of the TGP on climate has attracted worldwide attention. The primary purposes of this study were to determine the precipitation changes in the Three Gorges Reservoir Area and analyze the possible relationships with water level. We use the CN05.1 precipitation dataset, and water level data to analyze the variation characteristics of local precipitation in the TGRA.

Before we analyzed the variation characteristics of precipitation in the TGRA, the accuracy of CN05.1 in the 1° buffer zone of the TGRA was evaluated by using the site measured data provided by the Hubei Meteorological Bureau. The results show that the precipitation data of CN05.1 are of high accuracy. During the study period, the annual precipitation anomaly and the dry season precipitation anomaly show an increasing trend, while the flood season precipitation anomaly shows a slightly decreasing trend in the TGRA (*p* value > 0.05). The areas with a large change are mainly concentrated in the area near the TGD, both of which change from a decreasing trend to an increasing trend. These changes may be explained by the TGRA storage in the dry season and drainage in the flood season. The impounding causes the water level to rise and the water area to expand. The drainage decreases the water area, which affects the water vapor cycle and results in the changes in precipitation.

From 1998 to 2017, the spatial patterns of PCD in the TGRA are different, and the northeast is a particularly high-value area. The PCP shows a banding distribution, where the high-value area is also in northeast of TGRA. After the impoundment, the annual PCD and PCP values fluctuate less than before, and the average PCD value decreased. The PCP is delayed in the area around the TGD and Yichang Station. When the PCD is small, it is not conducive to the occurrence of flooding.

The CWT analysis indicates that the monthly average water level shows a significant positive correlation with the precipitation anomaly after 2011. No significant resonance phenomenon was found before. This phenomenon indicates that the higher water level may have a greater impact on local precipitation.

Our findings can provide some helpful information for the government and other departments to understand the climate impacts of large hydroelectric projects on localities. This study is mainly based on the comparison and analysis of precipitation and water level data. The impact mechanism of reservoirs on region climate warrants further discussed in future studies. In the TGRA, the precipitation anomalies in the flood season and dry season display different trends, which are likely resulting from the construction of the dam or other effects. The trends are also worthy of further research.

## Figures and Tables

**Table 1:** Statistical metrics summarizing the performance of monthly precipitation estimates relative to station observations (RMSE unit: mm/month for monthly data).

	January	February	March	April	May	June	July	August	September	October	November	December
CC	0.89	0.94	0.89	0.91	0.86	0.85	0.86	0.86	0.90	0.91	0.93	0.90
RMSE	6.43	8.07	11.88	20.48	30.27	42.63	53.74	45.75	33.42	18.85	11.97	5.84

**Figure A1:**
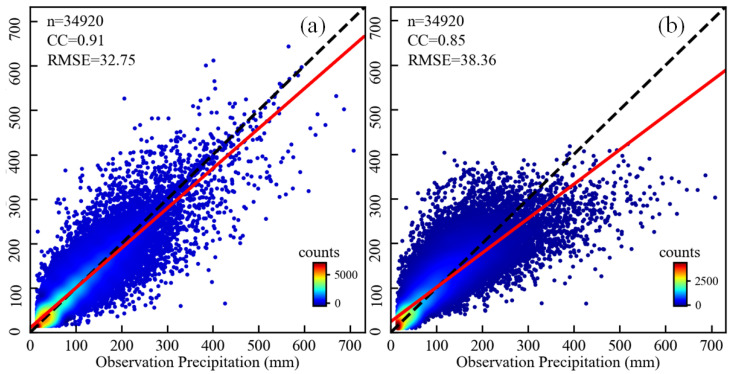
Density scatterplots of the monthly precipitation from (**a**) China Meteorological Forcing Dataset (CMFD) and (**b**) Peng et al. (2019) dataset versus ground-based observation. The black dashed line is the 1:1 line. n: valid number of samples; CC: Pearson correlation coefficient; RMSE: root-mean-square error of the monthly precipitation (mm); The color-coded dots represent the number of samples.

**Figure A2:**
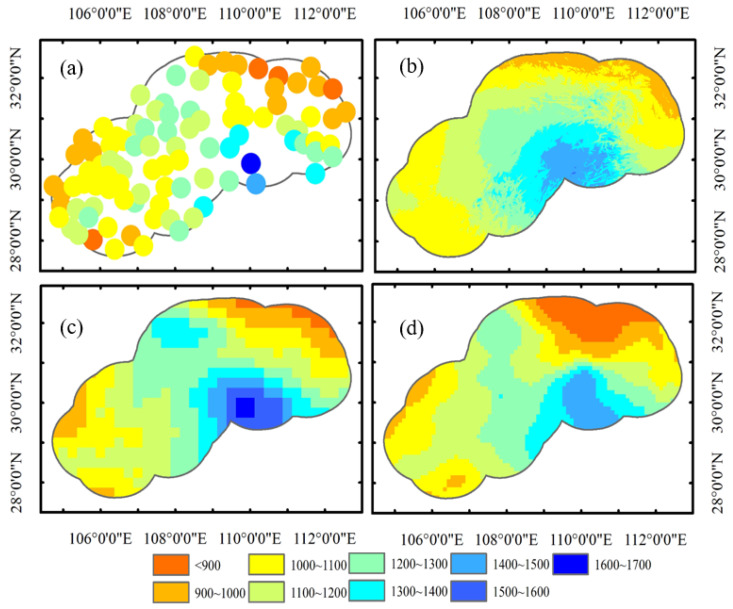
Spatial distribution of annual mean precipitation over 1988–2017 from (**a**) station observations, (**b**) Peng et al. (2019) dataset, (**c**) CN05.1, and (**d**) CMFD (unit: mm/yr).

## Data Availability

CN05.1 and station precipitation data are available upon request to the corresponding author. The daily water level data is available at (http://slt.hubei.gov.cn/sw/; accessed on 7 July 2021). China meteorological forcing dataset (CMFD) is available at (http://data.tpdc.ac.cn/en/data/8028b944-daaa-4511-8769-965612652c49/; accessed on 7 July 2021). 1-km monthly precipitation dataset for China is available at (https://data.tpdc.ac.cn/en/data/faae7605-a0f2-4d18-b28f-5cee413766a2/?q=1km; accessed on 7 July 2021).

## Appendix A. Comparison of Precipitation Forcing Data

We compared two other precipitation forcing data of China in the TGRA, including the 1 km monthly precipitation dataset for China and the China Meteorological Forcing Dataset (CMFD). The 1 km monthly precipitation dataset for China was spatially downscaled from the 300 Climatic Research Unit time-series dataset with the climatology dataset of WorldClim using delta spatial downscaling [53] (hereinafter, “Peng et al. (2019) dataset”). The CMFD is a near-surface meteorological and environmental reanalysis dataset provided by the Institute of Tibetan Plateau Research at the Chinese Academy of Science [54].

We evaluated the accuracy of these two datasets in the TGRA using the same method as in Section 3.1 (Figure A1 and Table A1). Figure A2 shows the spatial distributions of precipitation based on the three forcing datasets and station precipitation data. Results indicate that precipitation data from CN05.1 are more reliable in the TGRA.

**Table A1:** Statistical metrics summarizing the performance of monthly precipitation estimates based on different datasets relative to station observations (unit: mm/month for monthly data).

	CC		RMSE	
Time	Peng et al. (2019) dataset	CMFD	Peng et al. (2019) dataset	CMFD
January	0.77	0.66	9.29	14.15
February	0.88	0.83	10.96	14.03
March	0.74	0.77	17.51	19.91
April	0.75	0.87	31.84	24.08
May	0.69	0.82	42.17	34.80
June	0.60	0.82	64.23	47.99
July	0.63	0.82	80.90	60.54
August	0.68	0.81	64.94	52.97
September	0.75	0.86	51.03	36.75
October	0.77	0.84	27.22	24.15
November	0.80	0.84	19.92	18.89
December	0.77	0.67	8.86	12.36

In addition, we also used the three-cornered hat (TCH) method to evaluate the three datasets. Compared with the traditional error estimation methods, the TCH method can evaluate the accuracy of three or more different datasets when the true reference field is unknown [55,56]. In addition, this method is insensitive to the systematic biases and error cross-correlations contained within the datasets. The TCH method can be used to evaluate the uncertainty of multiple precipitation products in an iterative optimization process [57]. The RMSE values of CN05.1, CMFD and 1-km monthly precipitation dataset for China were obtained by the TCH method in the TGRA, which are 6.87 mm/month, 11.80 mm/month and 16.58 mm/month, respectively. CN05.1 has the lowest uncertainty in the TGRA, and the second lowest uncertainty is the CMFD, which adequately demonstrates the reliability of the CN05.1 dataset. Moreover, the TCH method is consistent with the evaluation method using station observations, and both show that CN05.1 has high accuracy in the TGRA.
